# Perineural invasion as an independent predictor of biochemical recurrence in prostate cancer following radical prostatectomy or radiotherapy: a systematic review and meta-analysis

**DOI:** 10.1186/s12894-018-0319-6

**Published:** 2018-02-01

**Authors:** Li-jin Zhang, Bin Wu, Zhen-lei Zha, Wei Qu, Hu Zhao, Jun Yuan, Ye-jun Feng

**Affiliations:** 1 Departments of Urology, Affiliated Jiang-yin Hospital of the Southeast University Medical College, Jiang-yin, 214400 China; 2 Departments of Pharmacy, Affiliated Jiang-yin Hospital of the Southeast University Medical College, Jiang-yin, 214400 China

**Keywords:** Perineural invasion, Prostate cancer, Radical prostatectomy, Radiotherapy, Biochemical recurrence, Meta-analysis

## Abstract

**Background:**

Although numerous studies have shown that perineural invasion (PNI) is linked to prostate cancer (PCa) risk, the results have been inconsistent. This study aimed to explore the association between PNI and biochemical recurrence (BCR) in patients with PCa following radical prostatectomy (RP) or radiotherapy (RT).

**Methods:**

According to the PRISMA statement, we searched the PubMed, EMBASE, Chinese National Knowledge Infrastructure (CNKI) and Wan Fang databases from inception to May 2017. Hazard ratios (HRs) and 95% confidence intervals (95% CIs) were extracted from eligible studies. Fixed or random effects model were used to calculate pooled HRs and 95% CIs according to heterogeneity. Publication bias was calculated by Begg’s test.

**Results:**

Ultimately, 19 cohort studies that met the eligibility criteria and that involved 13,412 patients (82-2,316 per study) were included in this meta-analysis. The results showed that PNI was associated with higher BCR rates in patients with PCa after RP (HR=1.23, 95% CI: 1.11, 1.36, *p*<0.001) or RT (HR=1.22, 95% CI: 1.12, 1.34, *p*<0.001). No potential publication bias was found among the included studies in the RP group (*p*-Begg = 0.124) or the RT group (*p*-Begg = 0.081).

**Conclusions:**

This study suggests that the presence of PNI by histopathology is associated with higher risk of BCR in PCa following RP or RT, and could serve as an independent prognostic factor in patients with PCa.

## Background

Prostate cancer (PCa) is the most common newly diagnosed cancer in males, with 1.6 million new cases per year; PCa is the third most common cause of cancer-related death in men [1]. Despite the use of radical prostatectomy (RP) and radiotherapy (RT) as initial therapies for localized PCa, approximately 18 % of patients eventually experience biochemical recurrence (BCR) [2]. Therefore, it is crucial to identify the patients who are at an increased risk of BCR after RP or RT. Preoperative prostate-specific antigen (p-PSA) levels [3], Gleason score (GS) [4] and pathological stage [5] are widely used as traditional risk factors for BCR. However, there is a growing interest in the identification of a new prognostic marker to improve the evaluation of the likelihood of BCR in PCa patients after local treatment.

Perineural invasion (PNI), which is considered a major mechanism for the extraprostatic spread of PCa [6], has been increasingly recognized as a novel prognostic marker [7]. However, whether PNI might be a prognostic factor for BCR is still under debate [8]. Some authors suggest that the presence of PNI is associated with adverse oncological outcomes and a higher risk of BCR, whereas others argue that PNI is not an independent predictor of BCR.

Therefore, to further clarify the relationship between PNI and the risk of BCR in PCa, we performed this systematic review and meta-analysis to evaluate whether the presence of PNI has a prognostic impact on BCR in patients following RP or RT.

## Methods

### Search strategy

According to the PRISMA guidelines [9], a systematic literature search of the PubMed, EMBASE, Chinese National Knowledge Infrastructure (CNKI) and Wan Fang databases was performed (up to May 2017). The search strategy used the following: (“prostate cancer” or “prostate AND neoplasms”) and (“radical prostatectomy” or “radiotherapy”) and (“perineural invasion”) and (“biochemical recurrence”). We also manually searched for potentially relevant studies from the references listed in the selected review articles. The language of the publications was limited to English and Chinese.

### Inclusion and exclusion criteria

The inclusion criteria for the eligible studies were as follows: (1) all patients were diagnosed with histologically confirmed PCa, and PNI in RP specimens and biopsy specimens were assessed by pathologists; (2) all patients underwent RP or RT; (3) BCR after RP was defined as a detectable or rising PSA value after surgery that was ≥0.2 ng/ml with a second confirmation (American Urological Association [10]) ; BCR after RT was defined as a rise in PSA level of ≥2 ng/ml above the nadir (American Society for Radiation Oncology and Radiation Therapy Oncology Group in Phoenix [11]); (4) the risk of BCR was estimated as hazard ratios (HRs) with corresponding 95% confidence intervals (CIs) or the risk could be calculated from the original articles; (5) the study was of a prospective or retrospective cohort design; (6) the articles were published in English or Chinese. The exclusion criteria were as follows: (1) letters, reviews, case reports, editorials and author responses; (2) non-human studies; (3) studies that did not analyze the outcome after PNI and BCR; (4) duplicate articles; (5) articles contained elements that were inconsistent with the inclusion criteria.

### Data extraction and Study Quality

The data of the eligible studies were extracted independently by two investigators (Zhen-lei Zha and Wei Qu). Any discrepancies were resolved by discussion with a third reviewer (Hu Zhao). The following data were extracted from the included studies: first author, year of publication, country, recruitment period, sample size, patient’s age, preoperative PSA level, Gleason score, pathological stage, positive percentage of PNI, definition of BCR, follow-up time and the HR(95%CI) of PNI for BCR. When the study provided the results of both the multivariate and univariate outcomes, we chose the multivariate model. The quality of the eligible studies was evaluated according to the Newcastle-Ottawa scale (NOS) [12], which include 3 domains with 8 items. Studies with scores of 7 or more stars were considered high- quality studies.

### Statistical analyses

All statistical analyses in this meta-analysis were performed by Stata 12.0 software (Stat Corp, College Station, TX, USA).The association between PNI and BCR outcome was presented as summary relative risk estimates (SRREs) and 95% CIs. Heterogeneity between studies was assessed using Q and I^2^ statistics. P < 0.10 or I^2^ > 50% was considered significant heterogeneity. A random effects model was used when obvious heterogeneity was observed, but otherwise, a fixed-effects model was used. Sensitivity analysis was used to estimate the reliability of the pooled results via the sequential omission of each study. Subgroup analyses were performed to examine potential sources of heterogeneity according to the adjusted parameters. Publication bias was assessed by funnel plots and was statistically determined by Begg’s tests; a *p* value < 0.05 was considered statistically significant.

## Results

### Search results

**Fig. 1:**
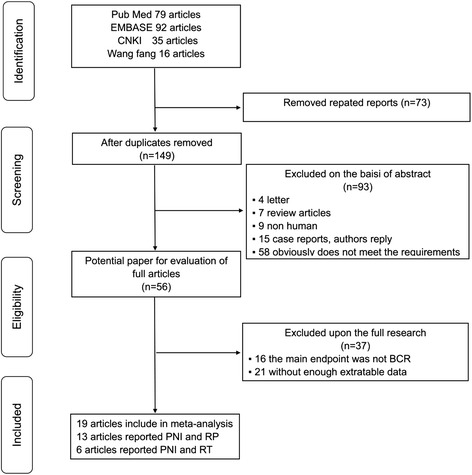
Flowchart of search and inclusion process for eligible studies

The search and selection process for eligible studies is shown in Fig. 1. In all, 222 articles were initially identified. Among them, 73 duplicates were excluded. After the abstracts were screened, 93 articles were excluded for the following reasons: non-human studies, letters, case reports, reviews, and other obvious irrelevant studies. A further 37 articles were excluded after full-text review because the results did not reported PNI on the BCR. Finally, 19 retrospective cohort studies met our inclusion criteria and were included in the meta-analysis. Of these, 13 studies with 10,807patients were analyzed to investigate whether PNI acts as a predictive biomarker of BCR in PCa following RP. Six studies with 2,605 patients were evaluated to determine the relationship between PNI and the risk of BCR in PCa following RT.

### Characteristics of the included studies

The main characteristics of the included studies are shown in Tables 1 and 2. All articles included were published in English, except for one, which was published Chinese [13]. All studies were published between 2003 and 2017, and the median duration of follow-up varied from 18.4 to 108 months. Patients in these studies were all diagnosed with PCa and received RP (13 studies) or RT (6 studies). Of the 19studies, 9 originated in Asia, and 10 were conducted in the other regions (USA, Australia, Belgium, Canada). This meta-analysis was based on a total sample size of 13,412 patients, of which 4,197 patients were reported to have PNI. Regarding the GS, 8,306 patients presented with a GS ≥7. For quality evaluated by the NOS, all the studies were found to be of high quality.

**Table 1:** Characteristics of the included studies on PNI in patients with PCa following RP

Author	Year	Country	Recruitment period	sample size	Age (years)	p-PSA (ng/ml)	GS≥7 (n.%)	Pathological stage ≥ pT2 (n.%)	PNI (n.%)	Follow-up (months)	NOS (score)
Ito et al. [32]	2003	Japan	1989-1998	82	Mean 66.5	Mean 17.2	47 (57.3%)	50(61%)	64 (78%)	Mean 21.7	7
Jeon et al. [25]	2009	Korea	1995-2004	237	Mean 64.5	Mean 11.2	183 (77.2%)	237(100%)	100 (42.2%)	Median 21.6	8
Lee et al. [18]	2010	Korea	1999-2010	361	Mean 69	Mean 15.6	160 (44.3%)	361(100%)	188 (52.1%)	Mean 42.2	7
Loeb et al. [33]	2010	USA	2002-2007	1256	Mean 56.1	Mean 5.7	293 (23.3%)	297(23.6%)	188 (15%)	Mean 33.6	8
Muramaki et al. [34]	2010	Japan	2003-2007	174	Mean 67.5	Mean 9.5	150 (86.2%)	128(73.6%)	107 (83.6%)	Median 44.3	7
Jung et al. [35]	2011	Korea	2005-2009	407	Mean 63.2	Mean 10.3	247 (60.7%)	307(75.4%)	129 (31.7%)	Median 18.4	7
Lee et al. [36]	2015	Korea	2011-2012	752	Mean 66	Mean 9.3	605 (80.5%)	260(34.6%)	483 (64.2%)	Mean 28.3	7
Reeves et al. [27]	2015	Australia	2005-2012	1497	Median 62	NR	1306 (87.2%)	1173 (78%)	1173 (78.4%)	Median 14	7
Watkins et al. [37]	2015	USA	2003-2010	279	Median 62	Median 5.5	80 (28.7%)	279(100%)	193 (69%)	Median 52.7	8
Zhu et al. [13]	2016	China	2002-2014	502	Median 67	Median 12.9	248 (49.4%)	502(100%)	411 (81.9%)	Median 28.6	7
Kang et al. [24]	2016	Korea	2003-2014	2034	Median 67	Median 7.4	1726 (84.9%)	NR	252 (12.4%)	Median 48	7
Kim et al. [38]	2016	Korea	2005-2016	2316	Median 65	Median 7.4	1621 (69.9%)	2316(100%)	NR	Median 60	8
Aoun et al. [5]	2017	Belgium	1990-2013	910	Mean 64	Median 6.8	189 (20.8%)	910(100%)	305 (33.5%)	Median 108	7

GS: Gleason score; PNI: perineural invasion; NR: not reported;NOS: Newcastle–Ottawa Scale; p-PSA: preoperative PSA

**Table 2:** Characteristics of the included studies on PNI in patients with PCa following RT

Author	Year	Country	Recruitment period	sample size	Age (years)	p-PSA (ng/ml)	GS≥7 (n.%)	PNI (n.%)	Radiotherapy	Follow-up (months)	NOS (score)
Copp et al[39]	2005	USA	1997-2000	93	Median 69.1	Median 9.1	34(36.6%)	17(18.3%)	EBRT	Median 45	7
Yu et al[19]	2007	USA	1993-2002	657	Mean 68	NR	238(36.2%)	112 (19.1%)	EBRT	Median 68	7
Pina et al[40]	2010	Canada	1999-2008	339	Mean 61.9	Mean 5.7	45(13.3%)	89(26.3%)	BT	Median 32	8
Feng et al[41]	2011	USA	1998-2008	718	Median 69.4	Median 8.3	474(66%)	220(34%)	EBRT	Median 62.2	8
Ding et al[30]	2012	USA	1998-2003	185	Median 69	NR	47(25.4%)	10(5%)	BT/ EBRT	Median 80	7
Spratt et al[42]	2013	USA	1989-2011	613	Median 70	NR	613(100%)	156(25.4%)	EBRT	Median 72	7

GS: Gleason score; PNI: perineural invasion; EBRT: external beam radiotherapy; BT: brachytherapy; NR: not reported; NOS: Newcastle–Ottawa Scale; p-PSA: preoperative PSA

### Relationship between PNI and BCR after RP

**Fig. 2:**
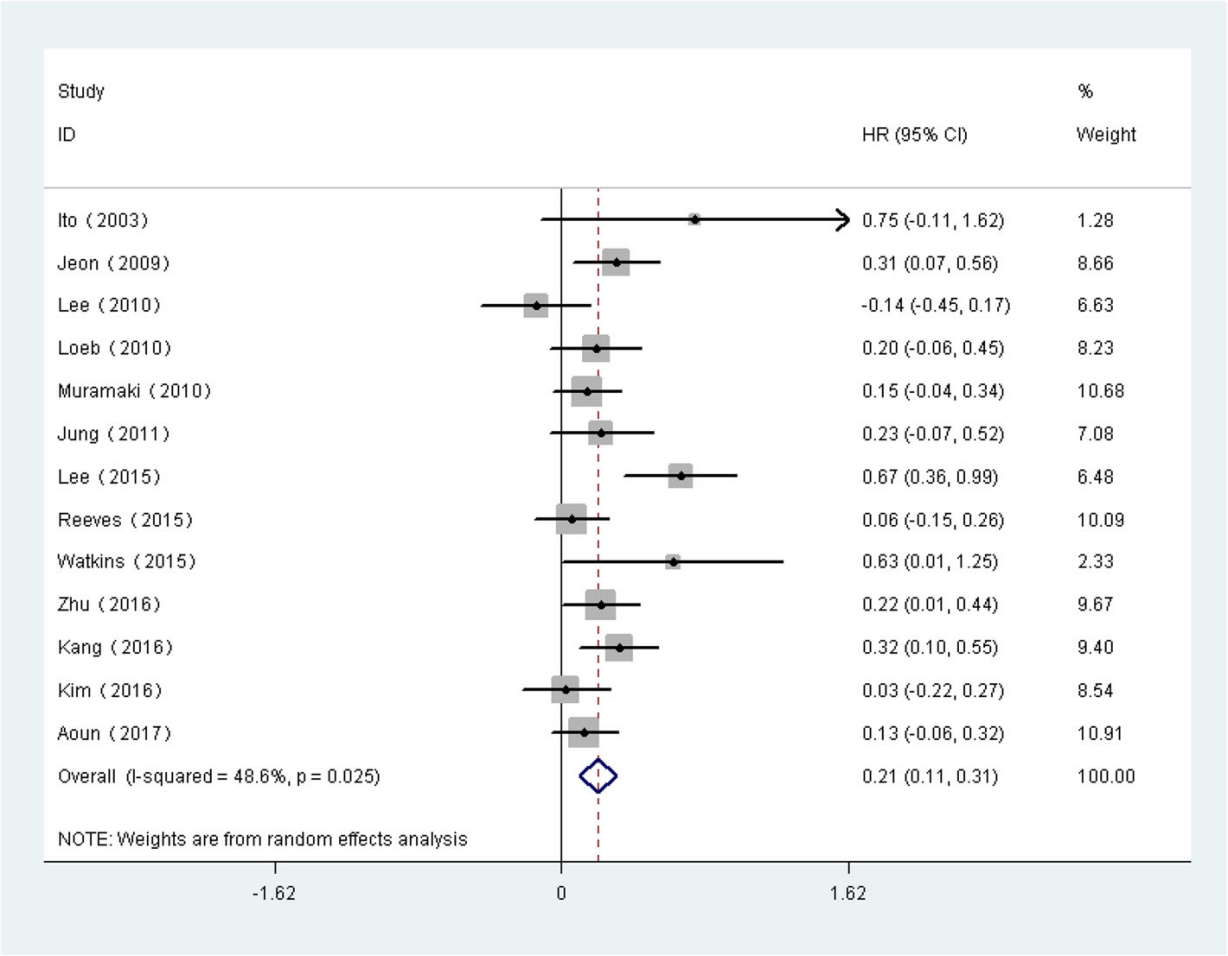
Forest plots for PNI and outcomes of BCR in PCa patients following RP

The forest plots of the meta-analyses are shown in Fig. 2. A random effects model was applied because the heterogeneity was evident among these studies (*p*= 0.025, I^2^= 48.6%). The pooled HR indicated that the presence of PNI was associated with a higher risk of BCR in patients with PCa after RP (HR=1.23, 95% CI: 1.11, 1.36, *p*<0.001). According to the sensitivity analysis, the SRRE ranged from 1.18 (95% CI: 1.09, 1.29) to 1.26 (95% CI: 1.14, 1.38) (Fig. 4a). In the subgroup analyses, we found that the heterogeneity was decreased significantly in some models, such as those of geographical region (“other regions”) and, age <65 years. It should also be noted that; the results of the subgroup analyses were consistent with the primary findings (Table 3). Furthermore, no statistical evidence of publication bias was found as assessed by Begg’s tests (*p* = 0.124) ( Fig. 5a).

**Table 3:** Summary and subgroup analyses of the eligible studies

Analysis specification	No. of studies	Study heterogeneity		Effects model	Pooled HR(95% CI)	*P*-Value
		I^2^(%)	*P*_heterogeneity_			
Radical prostatectomy						
Overall	13	48.6	0.025	Random	1.23 (1.11, 1.36)	<0.001
Geographical region						
Asia	9	57.8	0.013	Random	1.26 (1.10, 1.45)	0.001
Other regions	4	6	0.363	Fixed	1.23 (1.11, 1.36)	0.027
Age(years)						
≥65	7	67.2	0.006	Random	1.25 (1.04, 1.50)	0.015
<65	6	0	0.425	Fixed	1.20 (1.08, 1.32)	<0.001
p-PSA(ng/ml)						
≥10	4	44.4	0.126	Fixed	1.21 (1.01, 1.46)	0.037
<10	8	57.8	0.027	Random	1.28 (1.10, 1.49)	0.001
Sample size(cases)						
≥ 500	7	45.7	0.101	Fixed	1.23 (1.03, 1.47)	0.002
< 500	6	57.5	0.028	Random	1.24 (1.08, 1.41)	0.026
Median follow-up( months)						
≥ 30	7	34.9	0.162	Fixed	1.34 (1.12, 1.61)	0.001
< 30	6	57.9	0.036	Random	1.16 (1.11, 1.36)	0.014
Radiotherapy						
Overall	6	34.7	0.176	Fixed	1.22 (1.12, 1.34)	<0.001
Age(years)						
≥65	4	44.8	0.143	Fixed	1.21 (1.11, 1.33)	<0.001
<65	2	38.8	0.201	Fixed	1.45 (0.93, 2.24)	0.097
Sample size(cases)						
≥ 500	3	0	0.495	Fixed	1.20 (1.09, 1.31)	<0.001
< 500	3	31.0	0.235	Fixed	1.68 (1.18, 2.39)	0.004

### Association between PNI and BCR following RT

**Fig. 3:**
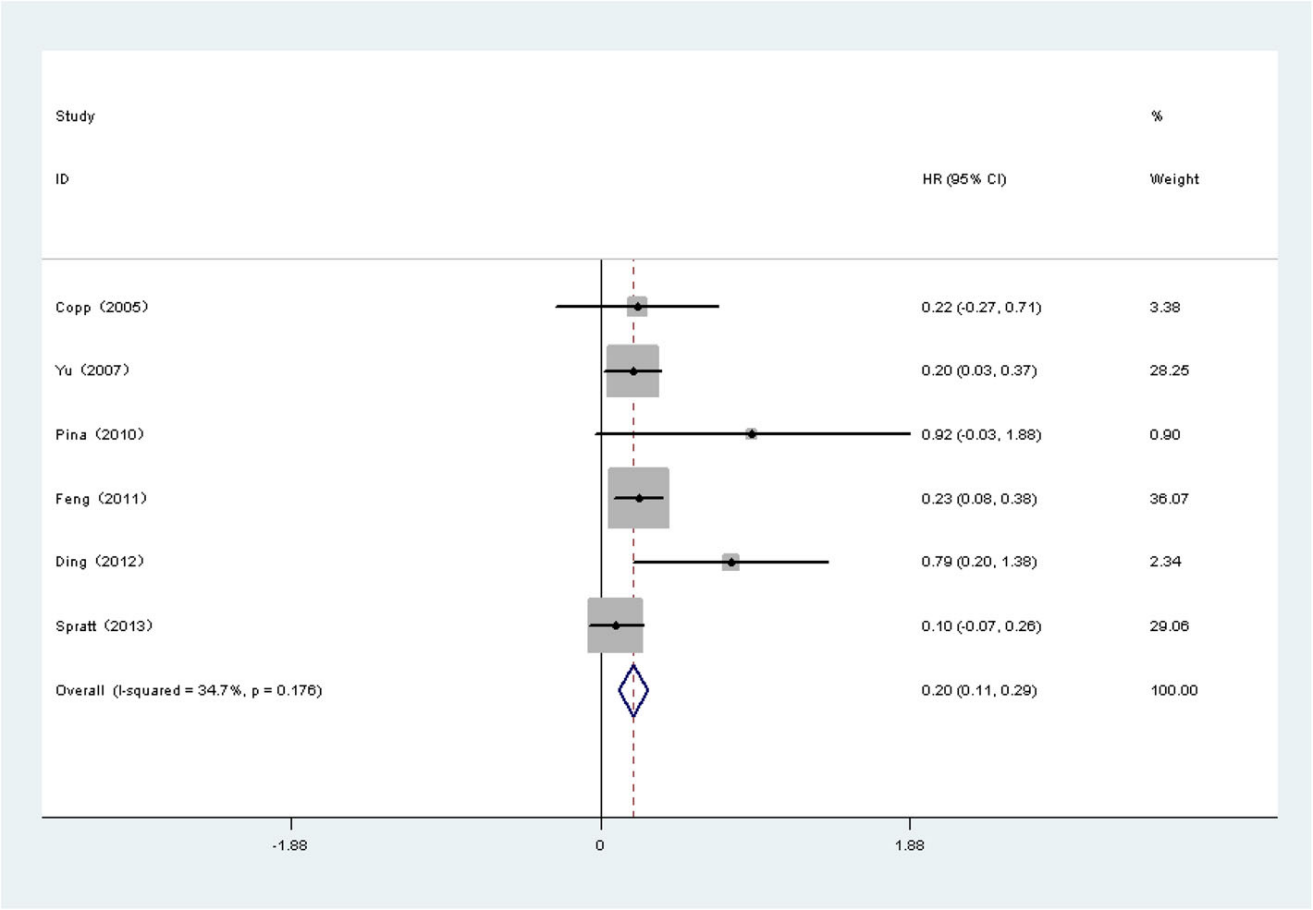
Forest plots for PNI and outcomes of BCR in PCa patients following RT

**Fig. 4:**
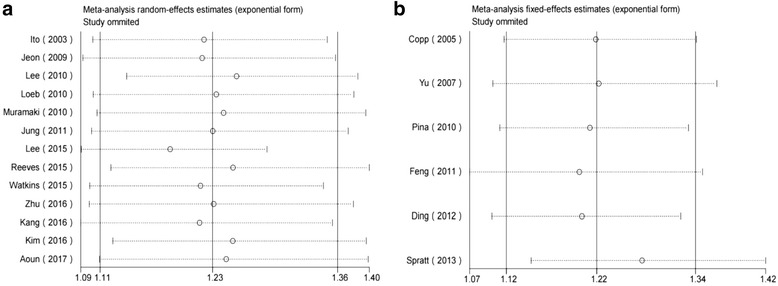
**a**: Sensitivity analysis for PNI and BCR in PCa following RP; **b**: Sensitivity analysis for PNI and BCR in PCa following RT

**Fig. 5:**
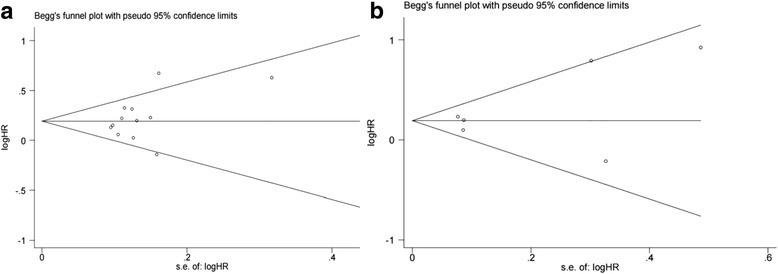
**a**: Funnel plots and Begg’s tests for the evaluation of publication bias in RP group; **b**: Funnel plots and Begg’s tests for the evaluation of publication bias in RT group

As shown in Fig. 3, the pooled HR from 6 studies indicated that PNI was an independent risk factor for BCA in PCa following RT (HR=1.22, 95% CI: 1.12, 1.34, *p*<0.001). Due to the lack of evidence of heterogeneity among the studies (*p*= 0.176, I^2^ = 34.7%), a fixed effects model was applied. The SRRE for BCR ranged from1.20 (95% CI: 1.07, 1.34) to 1.27(95% CI: 1.15, 1.42) according to the sensitivity analysis (Fig. 4b). The results of the subgroup analysis showed that PNI in a sample size ≥ 500 cases was significantly associated with BCR (n=3, HR=1.20, 95% CI: 1.09, 1.31, *p* <0.001) (Table 3). Begg’s tests (*p* = 0.081) provided no evidence of substantial publication bias in these studies (Fig. 5b).

## Discussion

With the wide spread use of the serum PSA level in PCa screening, most patients with newly diagnosed PCa present with clinically localized or locally advanced disease [14]. RP and RT have become the standard local treatments for localized PCa. However, approximately 1/3 of patients will develop BCR after RP, and 25~33% will experience BCR after RT [15]. Therefore, the characterization of the pathological features that may predict BCR is important for counseling patients and in directing the initial or adjuvant therapy. To date, seminal vesicle [16], surgical margins and extracapsular extension [17] are widely used as risk predictors of BCR in PCa. However, these are inferior and cannot accurately predict the risk of BCR in patients with PCa and complicated tumor backgrounds. Therefore, more sensitive and specific markers of risk are needed.

The phenomenon of peripheral nerve involvement has been overlooked for a long time, but it is now gaining recognition as a potential component of the cancer microenvironment [18]. PNI is defined as cancer cell tracking along or around a nerve within the perineural space [19]. It has been demonstrated that PNI may be a route of metastasis for many different cancers (pancreatic [20], bladder [21] and colorectal [22]). Patients with PNI have a higher risk of extraprostatic extension detected at the time of RP [23]; in other words, these patients have an increased risk of BCR.

However, the clinical significance of PNI in PCa following RP or RC is still controversial. Kang [24] and Jeon [25] showed that PNI is an adverse pathologic parameter and an independent predictor for BCR in PCa patients who undergo RP. Similarly, Yu [19] and Wong [26] considered that PNI is an independent risk factor associated with an increased risk of BCR in patients who undergo external beam radiotherapy. On the contrary, Reeves [27] and Freedland [28] reported that PNI is not correlated with extracapsular extension and BCR in PCa after RP. Nevertheless, Weight [29] suggested that the presence of PNI is not a significant predictor of BCR in patients undergoing brachytherapy (BT) for PCa, and Ding[30] demonstrated a significant independent association between PNI and an increased risk of biochemical failure in 185 PCa patients who received BT.

The conflicting results from different studies prompted us to perform this meta-analysis. For this study, we analyzed 19 studies that included 13,412 patients with PCa, of which 4,197 (31.2%) had PNI. Our results demonstrate that PNI was associated with a higher risk of BCR in PCa patients who underwent RP (p <0.001) or RT (p <0.001). These findings are similar to those reported by Kang [24] and Yu [19]. Notably, the overall findings in the present meta-analysis were consistently independent of geographical region, age, p-PSA, sample size, publication year, and follow-up in the RP group; the findings were also independent of age and sample size in the RT series. In the sensitivity analysis, we sequentially excluded each study, and the reliability and robustness of the results were confirmed. Moreover, no evidence of significant publication bias was found in this analysis according to Funnel plots and Begg’s tests. Although subgroup analyses were conducted in the RP and RT groups, significant heterogeneity still existed in some subgroups. The relevant results indicated that these factors were not potential sources of high heterogeneity.

Several potential limitations of our meta-analysis need to be addressed. First, all the included studies were retrospective cohort studies, and data extracted from those studies may have led to inherent potential bias as an unmeasured and uncontrolled confounder. Second, only papers in English and Chinese were selected, and thus some studies that were published in other languages may have otherwise been eligible. Therefore, both selection and publication bias are possible. Third, the pathological diagnosis of PCa and the detection method of PNI varied throughout the eligible studies. Collectively, these factors may promote significant heterogeneity. Fourth, substantial heterogeneity was identified across the studies, and although subgroup analyses were conducted to explore the source of the heterogeneity, heterogeneity still existed in certain categories of analysis. Thus, our results should be interpreted with caution.

## Conclusions

To conclude, despite the limitations listed above, this meta-analysis suggested the prognostic and clinicopathological importance of PNI in PCa. The results demonstrated that the presence of PNI is associated with a high risk of BCR whether the patient undergoes RP or RT. Since BCR has been reported to lead to distant metastasis and cancer death [31], we suggested that some patients with PCa and PNI may benefit from adjuvant local or systemic therapy. However, more randomized controlled trials with standardized methods and long-term follow-up are needed to verify our results.

## Abbreviations

BCR: Biochemical recurrence
CIs: Confidence intervals
HRs: Hazard ratios
NOS: Newcastle–Ottawa Scale
PCa: Prostate cancer
PNI: Perineural invasion
PSA: Prostate-specific antigen
RP: Radical prostatectomy
RT: Radiotherapy

## References

[1] Siegel RL, Miller KD, Jemal A (2017). Cancer Statistics, 2017. CA: a cancer journal for clinicians.

[2] Fossati N, Karnes RJ, Cozzarini C, Fiorino C, Gandaglia G, Joniau S, Boorjian SA, Goldner G, Hinkelbein W, Haustermans K (2016). Assessing the Optimal Timing for Early Salvage Radiation Therapy in Patients with Prostate-specific Antigen Rise After Radical Prostatectomy. European urology.

[3] Qi P, Tsivian M, Abern MR, Banez LL, Tang P, Moul JW, Polascik TJ (2013). Long-term oncological outcomes of men undergoing radical prostatectomy with preoperative prostate-specific antigen <2.5 ng/ml and 2.5-4 ng/ml. Urologic oncology.

[4] Kir G, Seneldir H, Gumus E (2016). Outcomes of Gleason score 3 + 4 = 7 prostate cancer with minimal amounts (<6%) vs >/=6% of Gleason pattern 4 tissue in needle biopsy specimens. Annals of diagnostic pathology.

[5] Aoun F, Albisinni S, Henriet B, Tombal B, Van Velthoven R, Roumeguere T (2017). Predictive factors associated with biochemical recurrence following radical prostatectomy for pathological T2 prostate cancer with negative surgical margins. Scandinavian journal of urology.

[6] Ayala GE, Dai H, Ittmann M, Li R, Powell M, Frolov A, Wheeler TM, Thompson TC, Rowley D (2004). Growth and survival mechanisms associated with perineural invasion in prostate cancer. Cancer research.

[7] Cohn JA, Dangle PP, Wang CE, Brendler CB, Novakovic KR, McGuire MS, Helfand BT (2014). The prognostic significance of perineural invasion and race in men considering active surveillance. BJU international.

[8] Haki Yuksel O, Urkmez A, Verit A: Can perineural invasion detected in prostate needle biopsy specimens predict surgical margin positivity in D'Amico low risk patients? Archivio italiano di urologia, andrologia : organo ufficiale [di] Societa italiana di ecografia urologica e nefrologica 2016, 88(2):89-92. 10.4081/aiua.2016.2.89 27377081

[9] Liberati A, Altman DG, Tetzlaff J, Mulrow C, Gotzsche PC, Ioannidis JP, Clarke M, Devereaux PJ, Kleijnen J, Moher D (2009). The PRISMA statement for reporting systematic reviews and meta-analyses of studies that evaluate health care interventions: explanation and elaboration. Journal of clinical epidemiology.

[10] Thompson IM, Valicenti RK, Albertsen P, Davis BJ, Goldenberg SL, Hahn C, Klein E, Michalski J, Roach M, Sartor O (2013). Adjuvant and salvage radiotherapy after prostatectomy: AUA/ASTRO Guideline. The Journal of urology.

[11] Roach M, Hanks G, Thames H, Schellhammer P, Shipley WU, Sokol GH, Sandler H (2006). Defining biochemical failure following radiotherapy with or without hormonal therapy in men with clinically localized prostate cancer: recommendations of the RTOG-ASTRO Phoenix Consensus Conference. International journal of radiation oncology, biology, physics.

[12] Stang A (2010). Critical evaluation of the Newcastle-Ottawa scale for the assessment of the quality of nonrandomized studies in meta-analyses. European journal of epidemiology.

[13] Zhu YJ, Wang YQ, Pan JH, Dong BJ, Xu F, Sha JJ, Xue W (2016). Huang YR: [Value of perineural invasion in prostatectomy specimen in the assessment on tumor progression and prognosis]. Zhonghua wai ke za zhi [Chinese journal of surgery].

[14] Lee DJ, Mallin K, Graves AJ, Chang SS, Penson DF, Resnick MJ, Barocas DA (2017). Recent Changes in Prostate Cancer Screening Practices and Epidemiology. J Urol.

[15] Fakhrejahani F, Madan RA, Dahut WL (2017). Management Options for Biochemically Recurrent Prostate Cancer. Current treatment options in oncology.

[16] Porcaro AB, de Luyk N, Corsi P, Sebben M, Tafuri A, Tamanini I, Processali T, Cerruto MA, Migliorini F, Brunelli M (2017). Bilateral lymph node micrometastases and seminal vesicle invasion associated with same clinical predictors in localized prostate cancer. Tumori.

[17] Psutka SP, Feldman AS, Rodin D, Olumi AF, Wu CL, McDougal WS (2011). Men with organ-confined prostate cancer and positive surgical margins develop biochemical failure at a similar rate to men with extracapsular extension. Urology.

[18] Lee JT, Lee S, Yun CJ, Jeon BJ, Kim JM, Ha HK, Lee W, Chung MK (2010). Prediction of perineural invasion and its prognostic value in patients with prostate cancer. Korean journal of urology.

[19] Yu HH, Song DY, Tsai YY, Thompson T, Frassica DA, DeWeese TL (2007). Perineural invasion affects biochemical recurrence-free survival in patients with prostate cancer treated with definitive external beam radiotherapy. Urology.

[20] Liang D, Shi S, Xu J, Zhang B, Qin Y, Ji S, Xu W, Liu J, Liu L, Liu C (2016). New insights into perineural invasion of pancreatic cancer: More than pain. Biochimica et biophysica acta.

[21] Muppa P, Gupta S, Frank I, Boorjian SA, Karnes RJ, Thompson RH, Thapa P, Tarrell RF, Herrera Hernandez LP, Jimenez RE (2017). Prognostic significance of lymphatic, vascular and perineural invasion for bladder cancer patients treated by radical cystectomy. Pathology.

[22] Zhou Y, Wang H, Gong H, Cao M, Zhang G, Wang Y (2015). Clinical significance of perineural invasion in stages II and III colorectal cancer. Pathology, research and practice.

[23] DeLancey JO, Wood DP, Jr., He C, Montgomery JS, Weizer AZ, Miller DC, Jacobs BL, Montie JE, Hollenbeck BK, Skolarus TA: Evidence of perineural invasion on prostate biopsy specimen and survival after radical prostatectomy. Urology 2013, 81(2):354-357. 10.1016/j.urology.2012.09.034 23374801

[24] Kang M, Oh JJ, Lee S, Hong SK, Lee SE, Byun SS (2016). Perineural Invasion and Lymphovascular Invasion are Associated with Increased Risk of Biochemical Recurrence in Patients Undergoing Radical Prostatectomy. Annals of surgical oncology.

[25] Jeon HG, Bae J, Yi JS, Hwang IS, Lee SE, Lee E (2009). Perineural invasion is a prognostic factor for biochemical failure after radical prostatectomy. International journal of urology : official journal of the Japanese Urological Association.

[26] Wong WW, Schild SE, Vora SA, Halyard MY (2004). Association of percent positive prostate biopsies and perineural invasion with biochemical outcome after external beam radiotherapy for localized prostate cancer. International journal of radiation oncology, biology, physics.

[27] Reeves F, Hovens CM, Harewood L, Battye S, Peters JS, Costello AJ, Corcoran NM (2015). Does perineural invasion in a radical prostatectomy specimen predict biochemical recurrence in men with prostate cancer?. Canadian Urological Association journal = Journal de l'Association des urologues du Canada.

[28] Freedland SJ, Csathy GS, Dorey F, Aronson WJ (2002). Percent prostate needle biopsy tissue with cancer is more predictive of biochemical failure or adverse pathology after radical prostatectomy than prostate specific antigen or Gleason score. The Journal of urology.

[29] Weight CJ, Ciezki JP, Reddy CA, Zhou M, Klein EA (2006). Perineural invasion on prostate needle biopsy does not predict biochemical failure following brachytherapy for prostate cancer. International journal of radiation oncology, biology, physics.

[30] Ding W, Lee J, Chamberlain D, Cunningham J, Yang L, Tay J (2012). Twelve-month prostate-specific antigen values and perineural invasion as strong independent prognostic variables of long-term biochemical outcome after prostate seed brachytherapy. International journal of radiation oncology, biology, physics.

[31] Pagano MJ, Whalen MJ, Paulucci DJ, Reddy BN, Matulay JT, Rothberg M, Scarberry K, Patel T, Shapiro EY, RoyChoudhury A (2016). Predictors of biochemical recurrence in pT3b prostate cancer after radical prostatectomy without adjuvant radiotherapy. Prostate.

[32] Ito K, Nakashima J, Mukai M, Asakura H, Ohigashi T, Saito S, Tachibana M, Hata J, Murai M (2003). Prognostic implication of microvascular invasion in biochemical failure in patients treated with radical prostatectomy. Urologia internationalis.

[33] Loeb S, Epstein JI, Humphreys EB, Walsh PC (2010). Does perineural invasion on prostate biopsy predict adverse prostatectomy outcomes?. BJU international.

[34] Muramaki M, Miyake H, Kurahashi T, Takenaka A, Fujisawa M (2010). Characterization of the anatomical extension pattern of localized prostate cancer arising in the peripheral zone. BJU international.

[35] Jung JH, Lee JW, Arkoncel FR, Cho NH, Yusoff NA, Kim KJ, Song JM, Kim SJ, Rha KH (2011). Significance of perineural invasion, lymphovascular invasion, and high-grade prostatic intraepithelial neoplasia in robot-assisted laparoscopic radical prostatectomy. Annals of surgical oncology.

[36] Lee D, Lee C, Kwon T, You D, Jeong IG, Hong JH, Ahn H, Kim CS (2015). Clinical features and prognosis of prostate cancer with high-grade prostatic intraepithelial neoplasia. Korean journal of urology.

[37] Watkins JM, Laszewski M, Watkins PL, Dufan TA, Adducci C (2015). Margin involvement at prostatectomy for clinically localized prostate cancer: does a low-risk group exist?. Practical radiation oncology.

[38] Kim DK, Koo KC, Abdel Raheem A, Kim KH, Chung BH, Choi YD, Rha KH (2016). Single Positive Lymph Node Prostate Cancer Can Be Treated Surgically without Recurrence. PLoS One.

[39] Copp H, Bissonette EA, Theodorescu D (2005). Tumor control outcomes of patients treated with trimodality therapy for locally advanced prostate cancer. Urology.

[40] Pina AG, Crook JM, Kwan P, Borg J, Ma C (2010). The impact of perineural invasion on biochemical outcome after permanent prostate iodine-125 brachytherapy. Brachytherapy.

[41] Feng FY, Qian Y, Stenmark MH, Halverson S, Blas K, Vance S, Sandler HM, Hamstra DA (2011). Perineural invasion predicts increased recurrence, metastasis, and death from prostate cancer following treatment with dose-escalated radiation therapy. International journal of radiation oncology, biology, physics.

[42] Spratt DE, Zumsteg Z, Ghadjar P, Pangasa M, Pei X, Fine SW, Yamada Y, Kollmeier M, Zelefsky MJ (2013). Prognostic importance of Gleason 7 disease among patients treated with external beam radiation therapy for prostate cancer: results of a detailed biopsy core analysis. International journal of radiation oncology, biology, physics.

